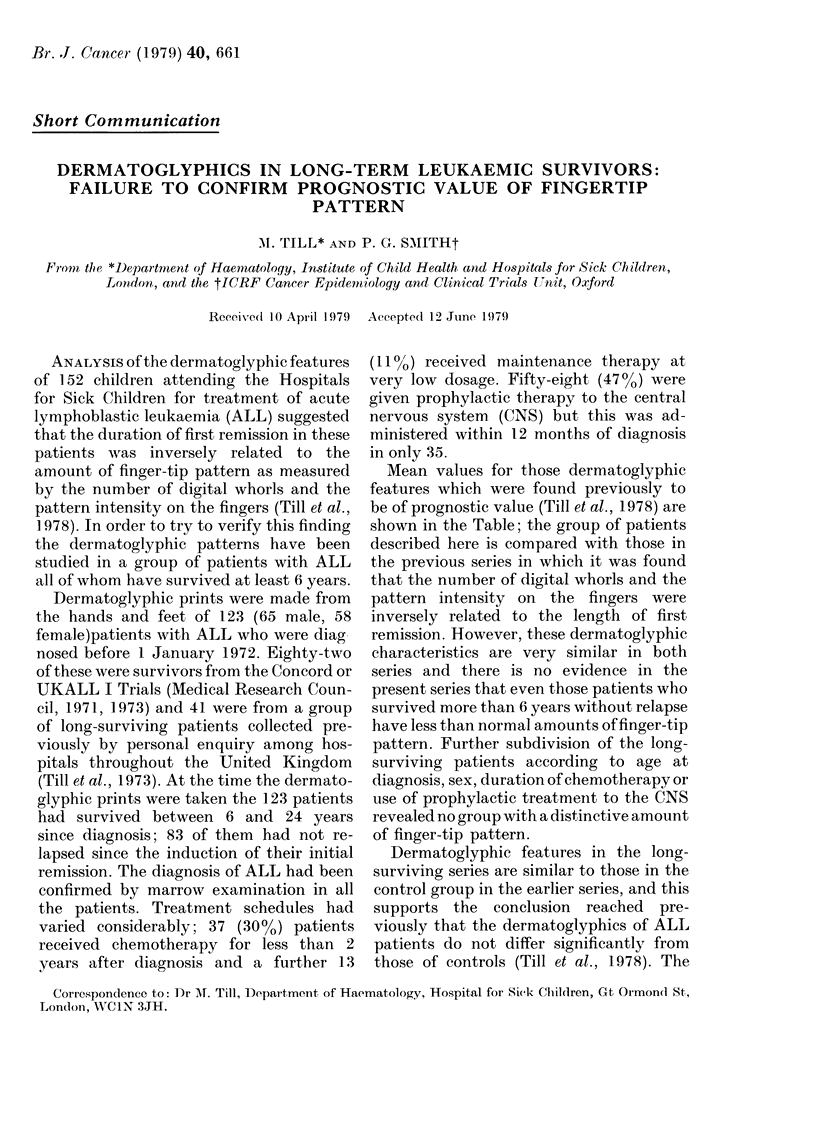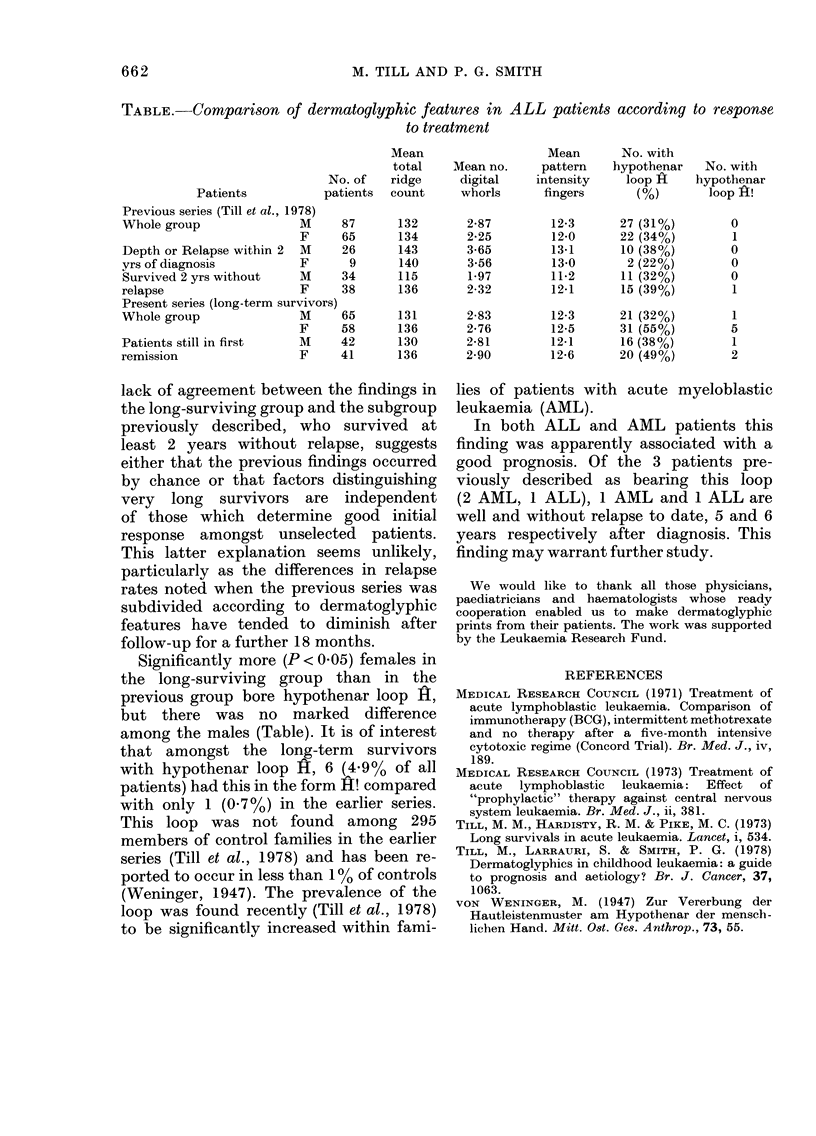# Dermatoglyphics in long-term leukaemic survivors: failure to confirm prognostic value of fingertip pattern.

**DOI:** 10.1038/bjc.1979.233

**Published:** 1979-10

**Authors:** M. Till, P. G. Smith


*Br. J. Cancer* (1979) **40**, 661

### *Short Communication*

### DERMATOGLYPHICS IN LONG-TERM LEUKAEMIC SURVIVORS: FAILURE TO CONFIRM PROGNOSTIC VALUE OF FINGERTIP PATTERN

M. TILL* AND P. G. SMITH†

*From the *Department of Haematology, Institute of Child Health and Hospitals for Sick Children, London, and the †ICRF Cancer Epidemiology and Clinical Trials Unit, Oxford*

Received 10 April 1979 Accepted 12 June 1979

ANALYSIS of the dermatoglyphic features of 152 children attending the Hospitals for Sick Children for treatment of acute lymphoblastic leukaemia (ALL) suggested that the duration of first remission in these patients was inversely related to the amount of finger-tip pattern as measured by the number of digital whorls and the pattern intensity on the fingers (Till *et al.*, 1978). In order to try to verify this finding the dermatoglyphic patterns have been studied in a group of patients with ALL all of whom have survived at least 6 years.

Dermatoglyphic prints were made from the hands and feet of 123 (65 male, 58 female)patients with ALL who were diagnosed before 1 January 1972. Eighty-two of these were survivors from the Concord or UKALL I Trials (Medical Research Council, 1971, 1973) and 41 were from a group of long-surviving patients collected previously by personal enquiry among hospitals throughout the United Kingdom (Till *et al.*, 1973). At the time the dermatoglyphic prints were taken the 123 patients had survived between 6 and 24 years since diagnosis; 83 of them had not relapsed since the induction of their initial remission. The diagnosis of ALL had been confirmed by marrow examination in all the patients. Treatment schedules had varied considerably; 37 (30%) patients received chemotherapy for less than 2 years after diagnosis and a further 13 (11%) received maintenance therapy at very low dosage. Fifty-eight (47%) were given prophylactic therapy to the central nervous system (CNS) but this was administered within 12 months of diagnosis in only 35.

Mean values for those dermatoglyphic features which were found previously to be of prognostic value (Till *et al.*, 1978) are shown in the Table; the group of patients described here is compared with those in the previous series in which it was found that the number of digital whorls and the pattern intensity on the fingers were inversely related to the length of first remission. However, these dermatoglyphic characteristics are very similar in both series and there is no evidence in the present series that even those patients who survived more than 6 years without relapse have less than normal amounts of finger-tip pattern. Further subdivision of the long-surviving patients according to age at diagnosis, sex, duration of chemotherapy or use of prophylactic treatment to the CNS revealed no group with a distinctive amount of finger-tip pattern.

Dermatoglyphic features in the long-surviving series are similar to those in the control group in the earlier series, and this supports the conclusion reached previously that the dermatoglyphics of ALL patients do not differ significantly from those of controls (Till *et al.*, 1978). The

Correspondence to: Dr M. Till, Department of Haematology, Hospital for Sick Children, Gt Ormond St, London, WC1N 3JH.

TABLE.—*Comparison of dermatoglyphic features in ALL patients according to response to treatment*

| Patients | | No. of patients | Mean total ridge count | Mean no. digital whorls | Mean pattern intensity fingers | No. with hypothenar loop Ĥ (%) | No. with hypothenar loop Ĥ! |
|---|---|---|---|---|---|---|---|
| Previous series (Till *et al.*, 1978) | | | | | | | |
| Whole group | M | 87 | 132 | 2·87 | 12·3 | 27 (31%) | 0 |
| | F | 65 | 134 | 2·25 | 12·0 | 22 (34%) | 1 |
| Depth or Relapse within 2 | M | 26 | 143 | 3·65 | 13·1 | 10 (38%) | 0 |
| yrs of diagnosis | F | 9 | 140 | 3·56 | 13·0 | 2 (22%) | 0 |
| Survived 2 yrs without | M | 34 | 115 | 1·97 | 11·2 | 11 (32%) | 0 |
| relapse | F | 38 | 136 | 2·32 | 12·1 | 15 (39%) | 1 |
| Present series (long-term survivors) | | | | | | | |
| Whole group | M | 65 | 131 | 2·83 | 12·3 | 21 (32%) | 1 |
| | F | 58 | 136 | 2·76 | 12·5 | 31 (55%) | 5 |
| Patients still in first | M | 42 | 130 | 2·81 | 12·1 | 16 (38%) | 1 |
| remission | F | 41 | 136 | 2·90 | 12·6 | 20 (49%) | 2 |

lack of agreement between the findings in the long-surviving group and the subgroup previously described, who survived at least 2 years without relapse, suggests either that the previous findings occurred by chance or that factors distinguishing very long survivors are independent of those which determine good initial response amongst unselected patients. This latter explanation seems unlikely, particularly as the differences in relapse rates noted when the previous series was subdivided according to dermatoglyphic features have tended to diminish after follow-up for a further 18 months.

Significantly more ($P < 0·05$) females in the long-surviving group than in the previous group bore hypothenar loop Ĥ, but there was no marked difference among the males (Table). It is of interest that amongst the long-term survivors with hypothenar loop Ĥ, 6 (4·9% of all patients) had this in the form Ĥ! compared with only 1 (0·7%) in the earlier series. This loop was not found among 295 members of control families in the earlier series (Till *et al.*, 1978) and has been reported to occur in less than 1% of controls (Weninger, 1947). The prevalence of the loop was found recently (Till *et al.*, 1978) to be significantly increased within families of patients with acute myeloblastic leukaemia (AML).

In both ALL and AML patients this finding was apparently associated with a good prognosis. Of the 3 patients previously described as bearing this loop (2 AML, 1 ALL), 1 AML and 1 ALL are well and without relapse to date, 5 and 6 years respectively after diagnosis. This finding may warrant further study.

We would like to thank all those physicians, paediatricians and haematologists whose ready cooperation enabled us to make dermatoglyphic prints from their patients. The work was supported by the Leukaemia Research Fund.

REFERENCES

MEDICAL RESEARCH COUNCIL (1971) Treatment of acute lymphoblastic leukaemia. Comparison of immunotherapy (BCG), intermittent methotrexate and no therapy after a five-month intensive cytotoxic regime (Concord Trial). *Br. Med. J.*, iv, 189.

MEDICAL RESEARCH COUNCIL (1973) Treatment of acute lymphoblastic leukaemia: Effect of "prophylactic" therapy against central nervous system leukaemia. *Br. Med. J.*, ii, 381.

TILL, M. M., HARDISTY, R. M. & PIKE, M. C. (1973) Long survivals in acute leukaemia. *Lancet*, i, 534.

TILL, M., LARRAURI, S. & SMITH, P. G. (1978) Dermatoglyphics in childhood leukaemia: a guide to prognosis and aetiology? *Br. J. Cancer*, **37**, 1063.

VON WENINGER, M. (1947) Zur Vererbung der Hautleistenmuster am Hypothenar der menschlichen Hand. *Mitt. Ost. Ges. Anthrop.*, **73**, 55.